# Impairments in recognition of emotional facial expressions, affective prosody, and multisensory facilitation of response time in high-functioning autism

**DOI:** 10.3389/fpsyt.2023.1151665

**Published:** 2023-04-24

**Authors:** Jonatan Hoffmann, Gabrielle Travers-Podmaniczky, Michael Alexander Pelzl, Carolin Brück, Heike Jacob, Lea Hölz, Anne Martinelli, Dirk Wildgruber

**Affiliations:** ^1^Department of General Psychiatry and Psychotherapy, University of Tübingen, Tübingen, Germany; ^2^School of Psychology, Fresenius University of Applied Sciences, Frankfurt am Main, Germany

**Keywords:** multisensory integration, facial expressions, affective prosody, high-functioning autism, emotional perception

## Abstract

**Introduction:**

Deficits in emotional perception are common in autistic people, but it remains unclear to which extent these perceptual impairments are linked to specific sensory modalities, specific emotions or multisensory facilitation.

**Methods:**

This study aimed to investigate uni- and bimodal perception of emotional cues as well as multisensory facilitation in autistic (*n* = 18, mean age: 36.72 years, SD: 11.36) compared to non-autistic (*n* = 18, mean age: 36.41 years, SD: 12.18) people using auditory, visual and audiovisual stimuli.

**Results:**

Lower identification accuracy and longer response time were revealed in high-functioning autistic people. These differences were independent of modality and emotion and showed large effect sizes (Cohen’s *d* 0.8–1.2). Furthermore, multisensory facilitation of response time was observed in non-autistic people that was absent in autistic people, whereas no differences were found in multisensory facilitation of accuracy between the two groups.

**Discussion:**

These findings suggest that processing of auditory and visual components of audiovisual stimuli is carried out more separately in autistic individuals (with equivalent temporal demands required for processing of the respective unimodal cues), but still with similar relative improvement in accuracy, whereas earlier integrative multimodal merging of stimulus properties seems to occur in non-autistic individuals.

## 1. Introduction

For successful social interaction, it is crucial to recognize the emotional state of our counterpart and to act accordingly. To identify the current emotional state of others, we rely on the understanding of emotional cues simultaneously conveyed *via* various communicational channels, including auditory (i.e., the tone of voice, also referred to as prosody) as well as visual (i.e., facial expressions, gestures) cues. This ability can, however, be compromised in certain conditions. Autism spectrum condition is characterized by persistent deficits in social communication and social interaction, restricted, repetitive patterns of behavior, interests and activities as well as atypical sensory processing (1).

Previous literature has reported that autistic people show considerable impairments in recognition of various nonverbal emotional cues, including facial expressions and prosody, compared to non-autistic people (NAP). In a recent meta-analysis, a large effect size (Hedges’ *g* = −0.80) was calculated for the average impairment across different types of nonverbal cues (2). Furthermore, this meta-analysis revealed moderate impairments in the processing speed within the autistic group (*g* = −0.61).

For a closer look at cue-specific deficits, a meta-analysis by Zhang (3), exclusively focusing on recognition of affective prosody, revealed significantly reduced identification accuracy in autistic individuals with a moderate-to-large effect size (Hedges’ *g* = −0.63) as well as large effects regarding prolonged reaction times (*g* = −1.35). Furthermore, cue-specific differences with a small-to-moderate effect also were identified for reduced accuracy of recognition of emotional facial expressions (4). Besides these impairments in processing unimodal cues, a reduced identification accuracy has also been observed when audiovisual emotional cues are presented. Impairments of audiovisual emotion recognition range from moderate (5) to large (6).

It should be noted, however, that the true effects in these meta-analyses might be partially overestimated due to a publication bias favoring studies reporting significant impairments (2, 3). Conclusions regarding the relevance of these deficits for everyday life might be further compromised since most of the previous studies focused on unimodal emotion recognition. The majority evaluated either perception of auditory cues (7–16) or perception of visual cues (17–28). Furthermore, visual cue studies have mainly implemented static, pictorial stimuli. These do not reflect typical perceptual processes in everyday life, which are normally characterized by multisensory dynamic nonverbal cues of interactional partners. Astonishingly few studies have addressed emotion recognition in autistic compared to non-autistic individuals based on dynamic audiovisual emotional cues (6, 29–33). Among these, studies are relatively inconsistent regarding their methodology, and the stimulus material is quite diverse. Some studies included body language (6) or verbal information (31) in the stimulus material, while others combined static visual stimuli with dynamic auditory stimuli (34), and still others focused exclusively on negative emotions (5). Thus, it remains unclear whether the degree of perceptual deficit is similar for prosody, facial expression, and bimodal audiovisual signals, or whether modality-specific or emotion-specific differences exist.

During perception of multisensory signals in healthy individuals, a facilitation of cerebral processing is reported, such that audiovisual cues can be recognized more accurately and faster than unimodal stimuli (35). The nomenclature for this effect varies, with the terms “multisensory facilitation” being used for multisensory facilitation of accuracy as well as facilitation of response time or, depending on the study, in some cases for parameters combining these two. In the current study, we focus on multisensory facilitation (MSF) of accuracy and multisensory facilitation of response time as two distinct parameters of multisensory integration.

Compared to non-autistic people, autistic individuals have been shown to benefit significantly less from multimodal presentation, in terms of both accuracy as well as response time, with an overall effect size of −0.41, indicating a small-to-moderate effect across various stimuli in a recent meta-analysis (36). Thereby, multisensory facilitation of response time has been frequently investigated using simple, non-social stimuli (e.g., beeps or flashes) and comparing response times to unimodal and audiovisual presentation. In these studies, autistic individuals showed less pronounced multisensory facilitation of response time than non-autistic people (37–40).

More complex, social stimuli have been used to investigate multisensory facilitation of accuracy, mainly by focusing on speech. In healthy individuals, audiovisual presentation of speech leads to a more accurate perception of the semantic language content (41). In contrast, autistic children benefit less from receiving speech information from multiple sensory modalities (42–46). Impairments in audiovisual speech integration in autistic individuals have furthermore been related to complex audiovisual integration at the cerebral level, with low-level integrational abilities reported to remain intact (47).

Based on these results, it can be assumed that deficits in multisensory facilitation also affect perception of nonverbal emotional cues in autistic people. This has been shown in a study with affective vocalizations and respective facial expressions, in which autistic individuals benefited significantly less from the presentation of audiovisual stimuli than non-autistic people (5) during disgust and fear processing. At the cerebral level, differences in multisensory visual and auditory nonverbal emotional cue processing in autistic individuals have also been reported (48), along with a potential modulatory role of attention (49).

In summary, evidence exists for differences in multisensory (emotional) cue processing between autistic and non-autistic people. However, it remains unclear to which extent the perceptual impairments in autistic people are linked to specific sensory modalities, specific emotions or multisensory facilitation. Therefore, in the current study, stimuli with high ecological validity were used to investigate unimodal and bimodal perception of emotional cues in autistic compared to non-autistic individuals. For this purpose, video recordings of actors were presented either bimodally (AV = emotional facial expression and affective prosody) or unimodally visually (V = mute presentation, i.e., facial expression only) or unimodally auditorily (A = audio track, i.e., prosody only).

Since autism describes a large group of people with great interindividual variability, we included only people diagnosed with high-functioning early childhood autism (ICD-10: F84.0) or Asperger-Syndrome (ICD-10: F84.5) and thereby focused our work on autistic individuals with no impairments in intellectual abilities, also referred to as high-functioning autism (HFA). Based on the evidence presented above we hypothesized that, compared to non-autistic individuals,

1. Autistic individuals show lower accuracies in emotion recognition for each of the three modalities;
2. Autistic individuals show longer response times for each of the three modalities;
3. Autistic individuals show reduced multisensory facilitation of accuracy rates;
4. Autistic individuals show reduced multisensory facilitation of response times.

Moreover, we carried out exploratory analyses to evaluate if the extent of perceptual impairment differs between specific sensory modalities and specific emotional categories in autistic individuals regarding accuracy rates and response times compared to non-autistic individuals.

## 2. Materials and methods

### 2.1. Participants

Eighteen autistic and 18 non-autistic people participated in this study. The autistic individuals were recruited from the special outpatient consultation service for autistic adults of the Department of General Psychiatry and Psychotherapy at the University of Tübingen. All autistic people were diagnosed with high-functioning early childhood autism (F84.0) or Asperger-Syndrome (F84.5) according to the ICD-10 criteria (50) by fully trained psychiatrists based on extensive clinical examination. This included a comprehensive anamnesis and evaluation of interactional behavior as well as structured self-rating instruments completed by autistic individuals, Autism-spectrum Quotient AQ (51), Empathy Quotient EQ (52), Multiple-choice Vocabulary Intelligence Test MWT-B (53), Beck Depression Inventory BDI (54), and questionnaires for parents or close relatives able to report firsthand about the participant’s behavior during the first decade of life, Social Responsiveness Scale SRS (55), Social Communication Questionnaire SCQ/FSK (56), and Marburg Rating Scale for Asperger’s Syndrome MBAS (57). EQ, SRS, SCQ/FSK, and MBAS were assessed only for autistic people during the diagnostic process to achieve high diagnostic confidence. Since these parameters were not assessed for non-autistic people, these scores are not reported here. BDI was assessed to evaluate whether symptoms of depression (known as a common comorbidity of autism) are present in our study cohort. The MWT-B was used as a measure to approximate IQ and the “Self-Report Emotional Intelligence Test” (SREIT (58)) was used as a measure of emotional intelligence to compare the self-estimated emotional intelligence with the results of our analyses.

Non-autistic people were recruited from the pool of employees at the Medical Center of the University of Tübingen and from their acquaintances. The non-autistic people were selected to match the autistic individuals in terms of age, gender, IQ and educational level. The comparison group was screened with the AQ questionnaire to confirm they were not autistic. All participants spoke German at the level of a native speaker, had normal or corrected to normal vision and hearing and had a sufficient level of everyday function to fulfill the tasks required in this study. No data on ethnicity were recorded. An overview of the assessed data is provided in Table 1.

**Table 1:** Participant characteristics.

Baseline characteristics	NAP, *n* = 18			HFA, *n* = 18			*p* value
	*M*	SD	Range	*M*	SD	Range	
Gender							
Male	14			14			
Female	4			4			
Age (years)	36.41	12.18	22–62	36.72	11.36	23–57	0.938
Years of education	11.78	1.59	9–13	11.72	1.78	9–13	0.922
IQ	117.47	18.45	94–145	120.31	18.62	92–145	0.663
SREIT	126.78	9.14	106–141	95.44	10.23	77–112	<0.001
BDI	2.00	2.54	0–10	13.78	10.45	3–37	<0.001
AQ	11.83	3.11	7–20	37.24	7.73	19–44	<0.001

NAP, non-autistic people; HFA, high-functioning autism; IQ, Intelligence Quotient; SREIT, Self-Report Emotional Intelligence Test; BDI, Beck Depression Inventory; AQ, Autism-spectrum Quotient.

### 2.2. Stimulus material

Short video and audio clips were used as stimulus material in which professional actors nonverbally conveyed information about their current emotional state (happy, alluring, neutral, angry, or disgusted). The four professional actors (2 female, 2 male) were asked to say one of four words, which were selected and balanced based on the results of a previous assessment of their valence and arousal (59–62) and each had a neutral meaning [Möbel = furniture, female actor; Gabel = fork, male actor; Zimmer = room, male actor; Objekt = object, female actor; mean valence scores ± SD: 4.9 ± 0.4 on a 9-point self-assessment Manikin scale (63)]. While speaking, the actors were instructed to express one of the five emotional states simultaneously *via* facial expressions and modulations of their tone of voice. Recordings from every actor were used for each emotional state, with the resulting stimulus material comprising 20 videos (4 actors × 5 emotional states). The videos were then edited in such a way that only the actor’s face could be seen on a black background in order to exclude any influence by environmental factors. The video recordings were presented to participants in three different modalities: with video and audio track (audiovisual = AV, i.e., emotional facial expression and affective prosody), only the video with muted audio (visual = V, i.e., facial expression only) or only the audio track without the video (auditory = A, i.e., prosody only), resulting in a total set of 60 stimuli (20 per modality). In previous studies, these stimuli were evaluated as reliable and valid for measures of emotion recognition abilities, since the emotional information expressed by the actors was identified correctly well above chance level for each stimulus (61, 64, 65). In the original validation with 30 healthy participants, the stimuli with the highest percentage of correct classifications in the AV condition were selected from a total set of 630 stimuli (61). The classification accuracy was 57% (A), 70% (V), and 86% (AV) (61). A subset of these stimuli (only the stimuli with words with neutral meaning) was used in the current study.

In the current study, we aimed to create a balanced task design with respect to the number of emotions with positive and negative valence, matched for their arousal level. To this end, we included two distinct negative emotional states that are characterized by a high degree of arousal (anger and disgust) and two different positive emotional states also characterized by a high arousal level (happiness and sexual interest). Alluring stimuli (expressing sexual interest) were selected as the second category of nonverbal cues with a positive valence due to their relevance in social interaction and the conceptual distinction from happy cues (60, 65), which otherwise build the only positive category within the concept of “basic emotions” according to Ekman and Friesen (66). Additionally, we included neutral nonverbal cues. During recording of alluring stimuli, the actors were asked to nonverbally communicate sexual interest in an inviting manner. Alluring stimuli were relatively uniform across actors with a soft and sustained intonation in the lower frequency spectrum, slow changing facial expressions, mostly with a slight smile and a slight widening of the palpebral fissure and a lifting of one or both eyebrows.

### 2.3. Experimental design

The stimuli were presented to participants sitting in a chair in front of a 17-inch flat screen monitor wearing headphones. The sound was presented using binaural headphones (Sennheiser HD 515; Sennheiser electronic GmbH & Co. KG, Wedemark-Wennebostel, Germany), the volume was individually adjusted to a comfortable level by each participant. The “Presentation” software (Neurobehavioral Systems Inc., Albany, CA, United States) was used to present the stimuli and record the responses. First, a scale with the five different emotional states, horizontally aligned, appeared on the screen for 1 s. Then, a fixation cross appeared in the middle off the screen accompanied by a simultaneously presented pure tone (302 Hz) to attract the participant’s attention. Subsequently, a random stimulus was presented in the audiovisual, visual or auditory modality. The scale showing the five different categories of emotional states reappeared on the screen after stimulus offset and the participants were instructed to select the emotional state that, in their intuitive opinion, was most likely expressed in the stimulus. To select the answer, the participants pressed one of the five horizontally adjacent buttons on a Cedrus RB-730 response pad (Cedrus Corporation, San Pedro, CA, United States), corresponding to the position of the emotions displayed on the screen. As soon as the participant responded, a short visual feedback (700 ms) of the selected answer was displayed and the answer could not be changed anymore. The response window (10 s duration) started with stimulus onset. The total duration of a single trial varied from 3.7 to 12.7 s depending on the stimulus duration and the individual response time.

In order to avoid answering bias, the horizontal position of the five different emotional states was permutated between participants. While neutral always remained in the middle, the other emotional states were distributed randomly, with the restriction that the two positive and the two negative emotional states always appeared on the same side, either right or left of the “neutral” response option. This resulted in eight different answer scales, which were changed between participants.

Each participant performed a short training session, consisting of 15 stimuli that were not part of the main experiment, to become familiar with the task before starting with the actual experiment. All 60 stimuli were then presented to each participant in a random order.

### 2.4. Data analysis

The main focus of data analysis lay on the accuracy of answers and the time until an answer was given (response time). Accuracy rates were calculated as the proportion of correct answers. Accuracy rates and response times were averaged for each modality and for each emotional category. One-sided independent *t*-tests were calculated for both accuracy rates and response times for all three modalities. Furthermore, Cohen’s *d* was calculated as a measure of effect size. This information can be helpful to estimate sample sizes in future studies regarding modality effects on emotion recognition by autistic individuals.

For further exploration of the effect of cue modality and emotional category on accuracy rates and response times, a mixed-model design analysis of variance (ANOVA) with modality (auditory, visual, and audiovisual) and emotional category (happy, alluring, neutral, angry, and disgusted) as within-subject factors and group (NAP, HFA) as the between-subject factor was calculated for accuracy rates as well as response times. The BDI was used as a covariate. ANOVA results were Greenhouse–Geisser-corrected.

In the current literature, the term “multisensory facilitation” is used for facilitation of accuracy (increase in identification rates) as well as for facilitation of response time (increase in processing speed) or, in some studies, for parameters combining these two different aspects. In the current study, we focused on multisensory facilitation of accuracy and multisensory facilitation of response time as two distinct parameters of multisensory integration. Multisensory facilitation for accuracy was operationalized as the percent improvement (MSF-A%) in accuracy rate after audiovisual presentation compared to the highest accuracy rate after unimodal presentation. The unimodal modality with the higher accuracy rate (visual or auditory) was determined individually per participant. Thus, MSF-A% was calculated as: [(audiovisual accuracy—max. Unimodal accuracy)/max. Unimodal accuracy] × 100. Similarly, multisensory facilitation for response time was operationalized as percent reduction (MSF-RT%) in response time after audiovisual compared to the fastest unimodal response time per participant. MSF-RT% was thereby calculated as: [(min. Unimodal RT—audiovisual RT)/min. Unimodal RT] × 100. Thus, positive values of the MSF-A% and MSF-RT% reflect an improvement (better accuracy rate or shorter response time) during audiovisual presentation compared to best unimodal presentation. These calculations have already been used in previous studies to quantify effects of multisensory stimulus presentation (5, 37, 40).

For the statistical analysis of the MSF-A% and the MSF-RT%, we used a one-sided independent *t*-test between groups (NAP, HFA). Pearson correlation coefficients among all psychometric variables are reported in the Supplementary material (Supplementary Tables S1, S2). All statistical data analyses were performed using IBM SPSS Statistics, Version 27. Significance levels were set at *p* < 0.05.

### 2.5. Ethical approval

The study was performed in accordance with the ethical principles expressed in the Declaration of Helsinki. Approval of the research protocol was granted by the Medical Ethics Committee at the University of Tübingen, Germany (#469/2013BO2). Written informed consent was obtained from all participants prior to the involvement in this research.

## 3. Results

### 3.1. Accuracy

To evaluate our first hypothesis, one-sided independent *t*-tests were calculated to compare the mean accuracy rates between both groups for all three modalities. In accordance with our hypothesis, significantly lower accuracy rates were observed in the HFA group compared to the NAP group for audiovisual, *t*(21.74) = 3.50, *p* = 0.001, visual, *t*(27.53) = 3.13, *p* = 0.002, and auditory, *t*(34) = 3.11, *p* = 0.002 modalities, with large effect sizes (all Cohen’s *d* > 1).

**Figure 1:**
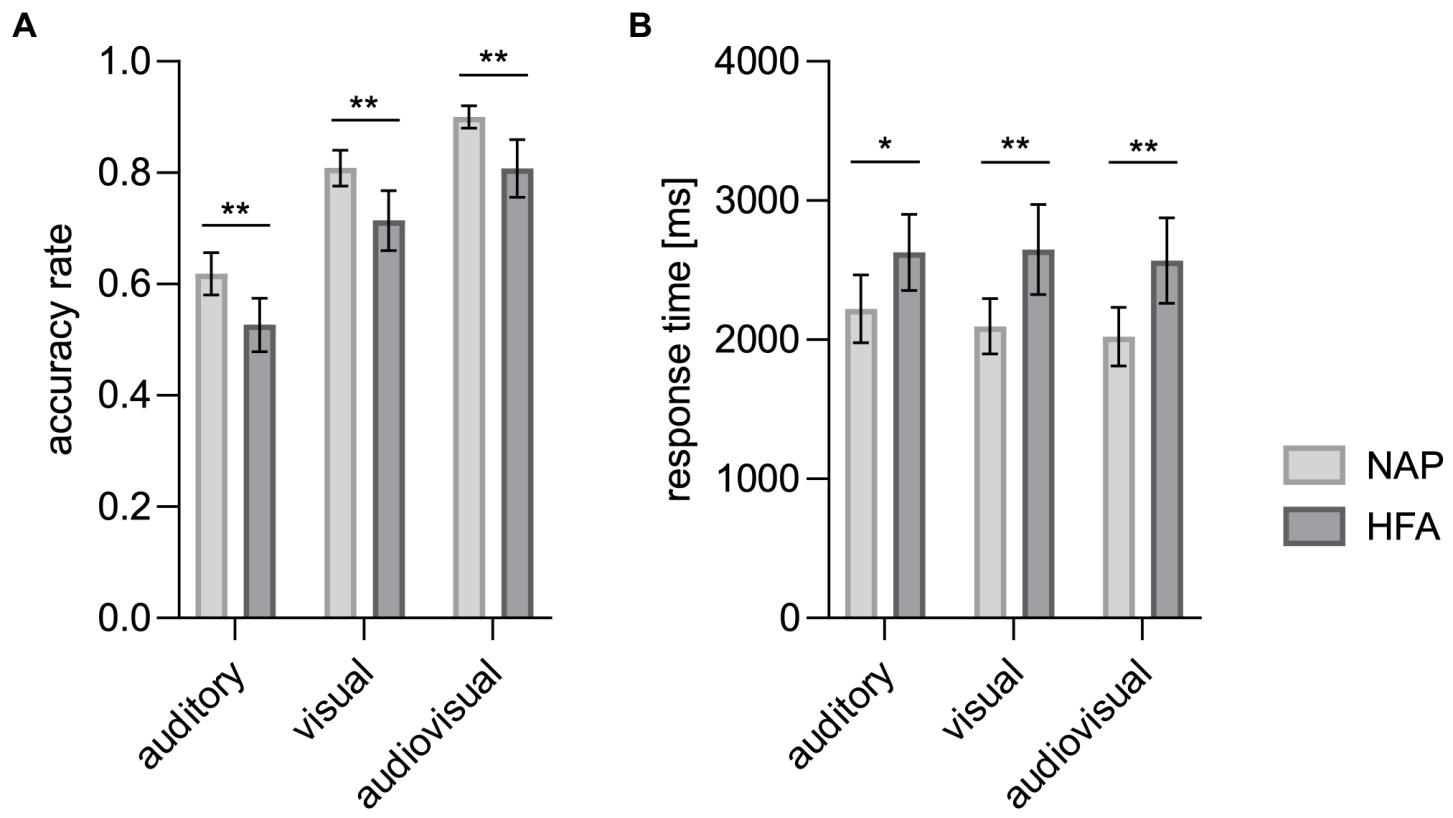
Mean accuracy rates **(A)** and mean response times **(B)** by modality. Significantly decreased accuracy rates and prolonged response times in autistic (HFA) as compared to non-autistic people (NAP) were observed for each of the three modalities. Error bars represent the corresponding 95%-CI. *p < 0.05. **p < 0.01.

Aiming to evaluate possible effects of cue modality and emotional category on the accuracy of emotion perception, an ANOVA was calculated in an exploratory approach. BDI was used as a covariate to evaluate whether it has a confounding effect on accuracy rates. The results revealed a significant main effect for group, *F*(1, 33) = 4.94, *p* = 0.033, partial *η*^2^ = 0.13, for cue modality, *F*(1.75, 57.72) = 98.61, *p* < 0.001, partial *η*^2^ = 0.75, and for emotional category, *F*(3.04, 100.44) = 18.12, *p* < 0.001, partial *η*^2^ = 0.16. Overall, accuracy rates were higher in NAP than in HFA. In both groups, the highest accuracy rates per modality were observed under audiovisual, followed by visual and auditory only modality (see Figure 1A; Table 2). In both groups, the highest accuracy rates per emotion were observed for neutral expressions and the lowest accuracy rates for disgusted expressions, while the accuracy rates for alluring, happy, and angry cues were in between (see Supplementary material; Supplementary Table S3).

**Table 2:** Accuracy rates (proportion of correct answers) per modality.

Modality	NAP		HFA		*p* value	Cohen’s *d*
	*M*	SD	*M*	SD		
Auditory	0.616	0.076	0.526	0.097	0.002	−1.04
Visual	0.807	0.064	0.714	0.109	0.002	−1.04
Audiovisual	0.899	0.040	0.806	0.105	0.001	−1.17
Overall	0.774	0.049	0.682	0.091	<0.001	−1.26

NAP, non-autistic people; HFA, high-functioning autism. *p*-values refer to one-sided independent *t*-tests between groups and Cohen’s *d* represent effect sizes of the group difference.

There was no statistically significant interaction between cue modality and group, *F*(1.75, 57.72) = 1.53, *p* = 0.227, partial *η*^2^ = 0.04, between emotional category and group, *F*(3.04, 100.44) = 1.01, *p* = 0.393, partial *η*^2^ = 0.03, or between cue modality, emotional category, and group, *F*(5.07, 167.32) = 1.03, *p* = 0.404, partial *η*^2^ = 0.03. There was also no significant effect of BDI, *F*(1, 33) = 1.45, *p* = 0.238, partial *η*^2^ = 0.04.

### 3.2. Response time

To evaluate our second hypothesis, one-sided independent *t*-tests were calculated to compare mean response times between both groups for each modality. These analyses showed significantly prolonged response times in the HFA group compared to the NAP group for audiovisual, *t*(34) = −3.11, *p* = 0.002, visual, *t*(34) = −3.08, *p* = 0.002, and auditory, *t*(34) = −2.35, *p* = 0.013, stimuli, with large effect sizes (all Cohen’s *d* > 0.8).

Additionally, an ANOVA was calculated to further explore effects of cue modality and emotional category on response times and BDI was again used as a covariate to evaluate whether it has a confounding effect on response rates. This exploratory approach revealed significant main effects for group, *F*(1, 33) = 6.42, *p* = 0.016, partial *η*^2^ = 0.16, for cue modality, *F*(1.48, 48.98) = 4.74, *p* = 0.021, partial *η*^2^ = 0.13, and for emotional category, *F*(2.83, 93.34) = 23.98, *p* < 0.001, partial *η*^2^ = 0.42. Overall, response times were prolonged in HFA compared to NAP. In both groups, the shortest mean response times per modality were observed under audiovisual modality (see Figure 1B; Table 3). In both groups, the shortest mean response times per emotion were observed for neutral expressions, followed by happy, angry, alluring, and disgusted expressions (see Supplementary material; Supplementary Table S4).

**Table 3:** Response times per modality.

Modality	NAP		HFA		*p* value	Cohen’s *d*
	*M* [ms]	SD [ms]	*M* [ms]	SD [ms]		
Auditory	2,222	488	2,629	550	0.013	0.78
Visual	2,096	397	2,648	648	0.002	1.03
Audiovisual	2,023	421	2,569	615	0.002	1.04
Overall	2,114	427	2,615	591	0.006	0.97

NAP, non-autistic people; HFA, high-functioning autism. *p*-values refer to one-sided independent *t*-tests between groups and Cohen’s *d* represent effect sizes of the group difference.

There was no statistically significant interaction between cue modality and group, *F*(1.48, 48.98) = 1.11, *p* = 0.322, partial *η*^2^ = 0.03, between emotional category and group, *F*(2.83, 93.34) = 0.60, *p* = 0.605, partial *η*^2^ = 0.02, or between cue modality, emotional category, and group, *F*(5.24, 173.07) = 0.62, *p* = 0.691, partial *η*^2^ = 0.02. There was also no significant effect of BDI, *F*(1, 33) = 0.20, *p* = 0.658, partial *η*^2^ = 0.01.

### 3.3. Multisensory facilitation

To evaluate the third and fourth hypotheses, we first calculated by one sample *t*-tests whether the multisensory facilitation of accuracy (MSF-A%) and response times (MSF-RT%) significantly differ from 0 for HFA and NAP each and compared MSF-A% and MSF-RT% between both groups using one-sided independent *t*-tests.

Thereby MSF-A% significantly differed from 0 for NAP, *t*(17) = 6.98, *p* = <0.001, as well as HFA, *t*(17) = 4.78, *p* = <0.001, meaning that multisensory facilitation of accuracy was observed in both groups. MSF-RT% differed significantly from 0 for NAP, *t*(17) = 2.54, *p* = 0.021, but not for HFA, *t*(17) = −0.866, *p* = 0.399, meaning that multisensory facilitation of response time is present in NAP but not HFA.

**Figure 2:**
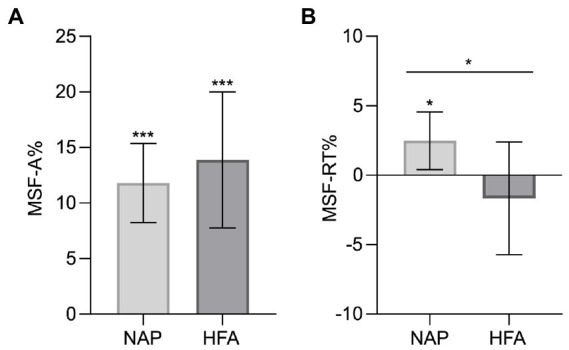
Multisensory facilitation (MSF) of accuracy **(A)** and of response time **(B)** showing a significant difference between NAP and HFA for the MSF of response time. Error bars represent the corresponding 95%-CI. MSF-A%, Multisensory facilitation of accuracy (percent improvement); MSF-RT%, Multisensory facilitation of response time (percent reduction); NAP, non-autistic people; HFA, high-functioning autism. *p < 0.05. **p < 0.01. ***p < 0.001.

Statistical analysis further revealed no significant group differences regarding MSF-A%, *t*(34) = −0.621, *p* = 0.270, Cohen’s *d* = −0.207, whereas significantly reduced MSF-RT% was observed in the HFA group, *t*(34) = 1.922, *p* = 0.032, Cohen’s *d* = 0.641. The HFA group showed a mean negative MSF-RT%, meaning that the average response times under audiovisual stimulus conditions tended to be even longer than under the respective fastest unimodal condition. This difference was, however, not significant. An overview of these data is shown in Figure 2 and Table 4.

**Table 4:** Multisensory facilitation of accuracy and response times.

Parameter	NAP		HFA		*p* value	Cohen’s *d*
	*M*	SD	*M*	SD		
MSF-A%	11.80	7.17	13.89	12.32	0.270	−0.207
MSF-RT%	2.48	4.16	−1.67	8.16	0.032	0.641

MSF-A%, Multisensory facilitation of accuracy (percent improvement); MSF-RT%, Multisensory facilitation of response time (percent reduction); NAP, non-autistic people; HFA, high-functioning autism. *p*-values refer to one-sided independent *t*-tests between groups and Cohen’s *d* represent effect sizes of the group difference.

Regarding unimodal conditions, the visual only modality was the fastest of the two unimodal stimulus conditions in 14 of the 18 participants (78%) in the NAP group, and in 10 of the 18 participants (56%) in the HFA group. This means that the proportion of participants with the auditory modality as the fastest unimodal modality was twice as high in the HFA group (8 of 18, 44%) than in the NAP group (4 of 18, 22%).

## 4. Discussion

The aim of this study was to investigate perception of auditory, visual, and audiovisual nonverbal emotional cues, as well as multisensory facilitation in autistic compared to non-autistic people.

In agreement with the literature, a significantly reduced accuracy rate was observed in autistic individuals for all three investigated modalities (auditory, visual, and audiovisual), thus confirming our first hypothesis. With our ecologically valid stimulus material, large effect sizes could be found in all three modalities (A: Cohen’s *d* = 1.04, V: Cohen’s *d* = 1.04, AV: Cohen’s *d* = 1.17). Moreover, no interaction between these group effects and modality- or emotion-specific differences were evident.

As a further aspect, in accordance with the literature, autistic people showed a significantly prolonged response time for each of the three modalities, meaning that our second hypothesis can also be confirmed. Again, we observed large effect sizes in all three modalities (A: Cohen’s *d* = 0.78, V: Cohen’s *d* = 1.03, AV: Cohen’s *d* = 1.04) and yet again, no interaction of group effects with modality- or emotion-specific differences emerged.

We further hypothesized that multisensory facilitation is reduced in autistic compared to non-autistic individuals in terms of both accuracy (Hypothesis 3) and response time (Hypothesis 4). However, we could only observe a significantly reduced multisensory facilitation for response time but not for accuracy, meaning that we can confirm Hypothesis 4 but did not find confirming evidence for Hypothesis 3 in our study. Particularly interesting is the fact that in contrast to the results obtained in the control group, we did not observe a significant multisensory facilitation of response times in autistic people at all. The mean MSF-RT% in the HFA group was in fact negative meaning that autistic people showed a tendency to even longer response times under audiovisual stimulus conditions compared to the respective unimodal stimuli, which was, however, not significant. This is in contrast with previous findings in which autistic individuals showed reduced multisensory facilitation of response times but still had facilitated response times as compared to unimodal presentation, albeit to a lesser degree than non-autistic people (37, 40). MSF-RT% showed a negative mean in the HFA group, although the mean response time in the HFA group was fastest in AV modality. This can be explained by the higher interindividual variability of MSF-RT% with stronger outliers (for positive as well as negative values) in HFA compared to NAP (see Supplementary material; Supplementary Figure S1; Supplementary Table S5) and by the observation that the auditory modality was more frequently the fastest of the two unimodal modalities in HFA compared to NAP (44% and 22% respectively).

The reduced multisensory facilitation of response times suggests that neural mechanisms underlying the integration of multisensory stimuli might differ in autistic compared to non-autistic people. Multisensory facilitation of response times in non-autistic people—as confirmed in numerous studies (36)—indicates a convergence of multisensory emotional cues to a bimodal percept at an early step of neural processing, which accelerates identification of the respective emotional state. The results of the current study indicate that in autistic individuals—in contrast—auditory and visual cues might be processed separately within modality-specific brain areas, whereas integration to a bimodal percept might occur to a lesser degree or occur at a later processing stage. These differences in the neural processing of multisensory cues might also contribute to the occurrence of specific symptoms such as sensitivity to light and noise or a general sensory overload in autistic people. However, it must be stated that this conclusion is speculative since the underlying neural mechanisms are not yet fully understood. On the one hand, neuro-oscillatory functions may be important for multisensory facilitation and seem to be altered in autistic people (67–70). Also a reduced inter-regional brain connectivity in autism (71, 72) could play a role, since it is essential that information from different sensory cortex areas are integrated into a joint percept in order to achieve successful multisensory integration. Further investigation is necessary for a better understanding of the underlying mechanisms.

Another very interesting observation is that, despite the reduced multisensory facilitation of response times, we did not find deficits in multisensory facilitation of accuracy (MSF-A% did not differ significantly between the groups). This suggests that autistic individuals perform a more time-consuming analysis of bimodal compared to unimodal emotional cues but can thereby still achieve the same relative improvement of accuracy. We suppose that this compensational strategy evolves during childhood development, since previous literature identified generally larger multisensory integration deficits in younger children than in adolescents (36) and particularly, the bimodal facilitation of accuracy in speech recognition (as a social stimulus) was impaired in autistic children (7–12 years) but not adolescents [13–15 years (43)].

Another explanation for the lack of difference in multisensory facilitation of accuracy in this current study might be the fact that no background noise or complex task demands were present in our experimental design. Prior studies of multisensory facilitation in speech recognition identified the largest impairments in autistic individuals when background noise was present at a level where non-autistic people benefited the most and the signal-to-noise ratio was low, while minor or even no deficits were observed without background noise (43, 45). For emotional signals, differences in multisensory integration were only found during complex (divided) attention conditions (49). Thus, in particular if compensational strategies are involved, it can be expected that differences in multisensory integration of emotional cues in autistic and non-autistic people become more pronounced with increasing task and non-task related demands. Experimental designs with increasing complexity likely approximate the simultaneous demands present in everyday interactional situations more accurately, and thereby may more precisely depict differences in multisensory integration experienced by autistic people. Thus, it would be interesting to further address the impact of background noise and complex task demands to multisensory integration of emotional cues in future research.

Some limitations of our study should be mentioned. Our experiment was only conducted using the described emotional stimuli. Other, non-emotional stimuli were not implemented. Therefore, we cannot distinguish whether the impairments of multisensory facilitation of response time in the HFA group are specifically related to emotion recognition or whether it rather reflects a general impairments in multisensory integration which has been observed in autistic individuals (37–40, 42–49). Although we implemented stimulus material with high ecological validity, each stimulus consisted only of one spoken word. As sentences in our daily life usually contain more than one word, they provide more prosodic information and facial expressions can be observed more intensely. Thus, emotion perception abilities might differ substantially in daily conversations. In addition, the male and female actors have spoken different words, so there might be a confounding influence of speaker gender and word content on the results. However, since the selected words had a neutral meaning and gender influences were not evaluated within this study, any influence would not be expected to systematically bias the reported results.

In summary, with our stimulus material, clear impairments in emotion perception are evident in autistic individuals. These impairments are independent of modality and emotion and show large effect sizes. Due to the reduced multisensory facilitation of response time with preserved multisensory facilitation of accuracy in the HFA group, it can be assumed that audiovisual stimuli might be analyzed separately (and thus more slowly) but still with the same relative improvement of accuracy in autistic individuals, whereas a more integrative stimulus perception seems to take place in non-autistic people. However, further investigation is still necessary to better understand the underlying neural mechanisms.

## Data availability statement

The raw data supporting the conclusions of this article will be made available by the authors, without undue reservation.

## Ethics statement

The studies involving human participants were reviewed and approved by Medical Ethics Committee at the University of Tübingen, Germany. The patients/participants provided their written informed consent to participate in this study.

## Author contributions

DW, CB, and HJ conceptualized the study. GT-P performed the data acquisition. JH, CB, GT-P, HJ, MP, LH, AM, and DW carried out the analysis and interpretation of the data. JH, DW, and AM carried out writing of the manuscript and preparation of the figures. All authors contributed to the manuscript revision, read and approved the submitted version, and provided approval for publication of the content.

## Funding

We acknowledge support by Open Access Publishing Fund of University of Tübingen.

## Conflict of interest

The authors declare that the research was conducted in the absence of any commercial or financial relationships that could be construed as a potential conflict of interest.

## Publisher’s note

All claims expressed in this article are solely those of the authors and do not necessarily represent those of their affiliated organizations, or those of the publisher, the editors and the reviewers. Any product that may be evaluated in this article, or claim that may be made by its manufacturer, is not guaranteed or endorsed by the publisher.
