# Supplementary material for: Impairments in recognition of emotional facial expressions, affective prosody, and multisensory facilitation of response time in high-functioning autism
Source: Front Psychiatry. 2023 Apr 24;14:1151665. doi: 10.3389/fpsyt.2023.1151665 (PMC10165112; doi:10.3389/fpsyt.2023.1151665)
Supplement: Supplementary file 1 [file Data_Sheet_1.docx]

Supplementary Material

Impairments in Recognition of Emotional Facial Expressions, Affective Prosody and Multisensory Facilitation of Response Time in High-Functioning Autism

Jonatan Hoffmann^*^, Gabrielle Travers-Podmaniczky, Michael Alexander Pelzl, Carolin Brück, Heike Jacob, Lea Hölz, Anne Martinelli and Dirk Wildgruber

*** Correspondence:** Jonatan Hoffmann: jonatanhoffmann1@gmail.com

# Supplementary correlation analysis

For descriptive purposes the Pearson correlation coefficients among all psychometric variables are presented in Table S1 and S2.

There was a strong correlation between Autism-spectrum Quotient (AQ) and Beck Depression Inventory (BDI, *r* = .707, *p* < .001) which indicates that participants who had a large autistic symptom severity also showed depressive symptoms. Using BDI as a covariate in our ANOVA analysis (see main text) revealed no significant effect, meaning that BDI has no confounding effect on the measured accuracy rates and response times.

There also was a strong (negative) correlation between AQ and overall accuracy rates (*r* = -.599, *p* < .001) and overall response times (*r* = .525, *p* < .001), indicating that participants with a large autistic symptom severity also showed lower accuracy rates and longer response times respectively.

The (negative) correlation between SREIT (Self-Report Emotional Intelligence Test) and overall accuracy rates (*r* = .418, *p* = .011) and overall response times (*r* = -.423, *p* = .010) indicates that participants could adequately self-estimate their actual emotion recognition abilities.

# Supplementary Tables

**Table S1**

*Overview of Pearson correlations with accuracy rate data for A, V, AV and Overall.*

| Variable | | AQ | BDI | SREIT | IQ | A | V | AV | Overall |
| --- | --- | --- | --- | --- | --- | --- | --- | --- | --- |
| BDI | *r* | .707^***^ |  |  |  |  |  |  |  |
|  | *p* | < .001 |  |  |  |  |  |  |  |
| SREIT | *r* | -.886^***^ | -.659^***^ |  |  |  |  |  |  |
|  | *p* | < .001 | < .001 |  |  |  |  |  |  |
| IQ | *r* | .185 | -.054 | -.177 |  |  |  |  |  |
|  | *p* | .303 | .766 | .323 |  |  |  |  |  |
| A | *r* | -.529^***^ | -.559^***^ | .395^*^ | .079 |  |  |  |  |
|  | *p* | < .001 | < .001 | .017 | .662 |  |  |  |  |
| V | *r* | -.511^**^ | -.376^*^ | .403^*^ | -.139 | .667^***^ |  |  |  |
|  | *p* | .002 | .024 | .015 | .439 | < .001 |  |  |  |
| AV | *r* | -.566^***^ | -.327 | .418^*^ | -.210 | .593^***^ | .824^***^ |  |  |
|  | *p* | < .001 | .051 | .011 | .242 | < .001 | < .001 |  |  |
| Overall | *r* | -.599^***^ | -.473^**^ | .454^**^ | -.099 | .847^***^ | .932^***^ | .898^***^ |  |
|  | *p* | < .001 | .004 | .005 | .585 | < .001 | < .001 | < .001 |  |
| MSF-A% | *r* | .105 | .251 | -.152 | -.074 | -.355^*^ | -.584^***^ | -.038 | -.374^*^ |
|  | *p* | .548 | .140 | .375 | .682 | .034 | < .001 | .825 | .025 |

AQ: Autism-spectrum Quotient; BDI: Beck Depression Inventory; SREIT: Self-Report Emotional Intelligence Test; IQ: Intelligence Quotient; A: Auditory; V: Visual; AV: Audiovisual; MSF-A%: Multisensory facilitation of accuracy (percent improvement). *: *p* < .05. **: *p* < .01. ***: *p* < .001.

**Table S2**

*Overview of Pearson correlations with response time data for A, V, AV and Overall.*

| Variable | | AQ | BDI | SREIT | IQ | A | V | AV | Overall |
| --- | --- | --- | --- | --- | --- | --- | --- | --- | --- |
| A | *r* | .412^*^ | .158 | -.338^*^ | -.003 |  |  |  |  |
|  | *p* | .014 | .358 | .044 | .989 |  |  |  |  |
| V | *r* | .553^***^ | .249 | -.400^*^ | .098 | .910^***^ |  |  |  |
|  | *p* | < .001 | .143 | .016 | .586 | < .001 |  |  |  |
| AV | *r* | .568^***^ | .249 | -.423^*^ | .144 | .932^***^ | .975^***^ |  |  |
|  | *p* | < .001 | .143 | .010 | .424 | < .001 | < .001 |  |  |
| Overall | *r* | .525^***^ | .225 | -.396^*^ | .083 | .966^***^ | .983^***^ | .990^***^ |  |
|  | *p* | < .001 | .188 | .017 | .644 | < .001 | < .001 | < .001 |  |
| MSF-RT% | *r* | -.479^**^ | -.217 | .333^*^ | -.379^*^ | -.217 | -.323 | -.454^**^ | -.341^*^ |
|  | *p* | .004 | .205 | .047 | .029 | .203 | .055 | .005 | .042 |

AQ: Autism-spectrum Quotient; BDI: Beck Depression Inventory; SREIT: Self-Report Emotional Intelligence Test; IQ: Intelligence Quotient; A: Auditory; V: Visual; AV: Audiovisual; MSF-RT%: Multisensory facilitation of response time (percent reduction). *: *p* < .05. **: *p* < .01. ***: *p* < .001.

**Table S3**

*Accuracy rates (proportion of correct answers) per emotional category.*

| Emotion | NAP | | HFA | | *p* | Cohen’s *d* |
| --- | --- | --- | --- | --- | --- | --- |
|  | *M* | *SD* | *M* | *SD* |  |  |
| Happy | .786 | .075 | .647 | .140 | .001 | -1.24 |
| Alluring | .840 | .088 | .668 | .243 | .010 | -.94 |
| Neutral | .859 | .092 | .852 | .106 | .836 | -.07 |
| Angry | .728 | .127 | .668 | .144 | .195 | -.44 |
| Disgusted | .658 | .130 | .576 | .087 | .035 | -.74 |

NAP: non-autistic people; HFA: high-functioning autism. *p*-values refer to two-sided independent t-tests between groups and Cohen’s *d* represent effect sizes of the group difference.

**Table S4**

*Response times per emotional category.*

| Emotion | NAP | | HFA | | *p* | Cohen’s *d* |
| --- | --- | --- | --- | --- | --- | --- |
|  | *M* [ms] | *SD* [ms] | *M* [ms] | *SD* [ms] |  |  |
| Happy | 1855 | 375 | 2375 | 635 | .005 | 1.00 |
| Alluring | 2323 | 443 | 2879 | 653 | .005 | 1.00 |
| Neutral | 1806 | 388 | 2350 | 598 | .003 | 1.08 |
| Angry | 2100 | 444 | 2520 | 562 | .018 | .83 |
| Disgusted | 2483 | 615 | 2953 | 712 | .041 | .71 |

NAP: non-autistic people; HFA: high-functioning autism. *p*-values refer to two-sided independent t-tests between groups and Cohen’s *d* represent effect sizes of the group difference.

**Table S5**

*Descriptive statistics of MSF-RT%.*

| Parameter | | NAP | HFA |
| --- | --- | --- | --- |
| Mean | | 2.48 | -1.67 |
| 95% CI for Mean | Lower Bound | .42 | -5.72 |
|  | Upper Bound | 4.55 | 2.39 |
| Median | | 2.59 | -.96 |
| Variance | | 17.28 | 66.62 |
| Std. Deviation | | 4.16 | 8.16 |
| Minimum | | -4.30 | -19.94 |
| Maximum | | 8.91 | 11.39 |
| Range | | 13.20 | 31.33 |
| Interquartile Range | | 7.48 | 11.08 |

NAP: non-autistic people; HFA: high-functioning autism.

# Supplementary Figures

**Supplementary Figure S1.** Boxplots of MSF-RT% in NAP and HFA group. MSF-RT%: Multisensory facilitation of response time (percent reduction); NAP: non-autistic people; HFA: high-functioning autism.
